# Transcriptional dysregulation of the *p73L* / *p63* / *p51* / *p40* / *KET* gene in human squamous cell carcinomas: expression of **Δ** Np73L, a novel dominant-negative isoform, and loss of expression of the potential tumour suppressor p51

**DOI:** 10.1054/bjoc.2000.1735

**Published:** 2001-05

**Authors:** M Senoo, I Tsuchiya, Y Matsumura, T Mori, Y Saito, H Kato, T Okamoto, S Habu

**Affiliations:** Department of Immunology,Department of Surgery, Tokai University School of Medicine, Bousei-dai, Isehara, Kanagawa 259-1193, Japan; Department of Molecular Genetics, Nagoya City University Medical School, Mizuho-cho, Mizuho-ku, Nagoya 467-0001, Japan; Laboratory of Biochemistry and Molecular Biology, The Rockefeller University, 1230 York Avenue, New York, NY 10021, USA; Core Research for Evolutional Science and Technology (CREST), Japan Science and Technology Corporation, Japan

**Keywords:** p53 homologue, p73L, ΔNp73L, squamous cell carcinoma

## Abstract

We have recently identified a second *p53* -related *p73L* gene, also referred to as *p63* / *p51* / *p40* / *KET* gene, which encodes the 2 major isoforms p73L and p51 resulting from different exon usage at their amino terminal regions. Although p73L and p51 are suspected to play oncogenic and tumour suppressive roles in mammalian cells, respectively, no evidence of linkage between the expression of these isoforms and human cancers has been reported so far. In this study, we first investigated the expression profile of p51 and p73L in various human tumour cell lines and found that a novel isoform, termed ΔNp73L, was predominantly expressed in squamous cell carcinomas. The expression profile of ΔNp73L/p73L/p51 in primary human skin cancer specimens showed that the expression of p51 was frequently lost (62%) but was detected in all normal skin samples. In p51-expressing skin cancers, ΔNp73L expression was associated at a high frequency (75%) though it was not detected in normal skin tissues. Transient co-transfection data indicate the possibility that ΔNp73L can inhibit p53-, and more preferentially, p51-mediated transactivation. These data suggest that the loss of expression of p51 and/or the expression of ΔNp73L might contribute to the pathogenesis of human squamous cell carcinomas. © 2001 Cancer Research Campaign http://www.bjcancer.com


British Journal of Cancer (2001) **84**(9), 1235–1241
© 2001 Cancer Research Campaign
doi: 10.1054/ bjoc.2001.1735, available online at http://www.idealibrary.com on IDEAL®

http://www.bjcancer.com

### Transcriptional dysregulation of the *p73L / p63 / p51 / p40 / KET* gene in human squamous cell carcinomas: expression of ΔNp73L, a novel dominant-negative isoform, and loss of expression of the potential tumour suppressor p51

**M Senoo[1,5], I Tsuchiya[2], Y Matsumura[1], T Mori[3], Y Saito[2], H Kato[4], T Okamoto[3] and S Habu[1,5]**

[1]Department of Immunology, [2] Department of Surgery, Tokai University School of Medicine, Bousei-dai, Isehara, Kanagawa 259-1193, Japan; [3]Department of Molecular Genetics, Nagoya City University Medical School, Mizuho-cho, Mizuho-ku, Nagoya 467-0001, Japan; [4]Laboratory of Biochemistry and Molecular Biology, The Rockefeller University, 1230 York Avenue, New York, NY 10021, USA; [5]Core Research for Evolutional Science and Technology (CREST), Japan Science and Technology Corporation, Japan

**Summary** We have recently identified a second *p53*-related *p73L* gene, also referred to as *p63*/*p51*/*p40*/*KET* gene, which encodes the 2 major isoforms p73L and p51 resulting from different exon usage at their amino terminal regions. Although p73L and p51 are suspected to play oncogenic and tumour suppressive roles in mammalian cells, respectively, no evidence of linkage between the expression of these isoforms and human cancers has been reported so far. In this study, we first investigated the expression profile of p51 and p73L in various human tumour cell lines and found that a novel isoform, termed ΔNp73L, was predominantly expressed in squamous cell carcinomas. The expression profile of ΔNp73L/p73L/p51 in primary human skin cancer specimens showed that the expression of p51 was frequently lost (62%) but was detected in all normal skin samples. In p51-expressing skin cancers, ΔNp73L expression was associated at a high frequency (75%) though it was not detected in normal skin tissues. Transient co-transfection data indicate the possibility that ΔNp73L can inhibit p53-, and more preferentially, p51-mediated transactivation. These data suggest that the loss of expression of p51 and/or the expression of ΔNp73L might contribute to the pathogenesis of human squamous cell carcinomas. © 2001 Cancer Research Campaign http://www.bjcancer.com

**Keywords**: p53 homologue; p73L; ΔNp73L; squamous cell carcinoma

Abnormalities of the *p53* tumour suppressor gene are among the most prevalent molecular events, and they contribute to tumorigenesis in over 50% of all human cancers (Hollstein et al, 1991; Levine et al, 1991; Greenblatt et al, 1994). However, despite the widespread presence of p53 mutations in human cancers, many tumours develop in the absence of p53 abnormalities or obvious tumour-specific antigens (Weinberg, 1993), most likely due to the involvement of other mechanisms of inactivating p53 or the loss of other tumour suppressor genes.

The first p53-related proteins, p73α and β, have been reported by Kaghad et al to be potentially invovled in the development of neuroblastoma (Kaghad et al, 1997). Jost et al have reported that p73 can induce apoptosis in tumour cells when overexpressed in a p53-independent manner (Jost et al, 1997). Laurenzi et al and Ueda et al have recently identified other p73 splice variants, p73γ, δ and ε, and suggested that these five p73 isoforms may differentially modulate the functions of p53 and p73 molecules (Laurenzi et al, 1998; Ueda et al, 1999). It is important to note that some of the regulatory mechanisms of p53 are implicated in the regulation of p73 functions by distinct mechanisms as revealed by tumour-derived mutant p53 (Di Como et al, 1999), mdm2 oncoprotein (Dobbelstein et al, 1999; Zeng et al, 1999) or viral oncoproteins (Marin et al, 1998; Prabhu et al, 1998; Steegenga et al, 1999). While proof of the biological importance of p73 has yet to be provided, it is noteworthy that *p53* is not an orphan gene but has a family with structural and functional resemblance like other tumour suppressors such as the retinoblastoma gene family (RB-1, p107 and p130) and the $p16^{INK4A}$-like cyclin-dependent kinase (cdk) inhibitors ($p15^{INK4B}$, $p16^{INK4A}$, $p18^{INK4C}$ and $p19^{INK4D}$).

Recently, we and other groups have identified the second *p53*-related gene, *p73L/p63/p51/p40/KET*, and cytogenetically mapped it in chromosome region 3q27–8 (Schmale and Banberger, 1997; Augustin et al, 1998; Osada et al, 1998; Senoo et al, 1998; Trink et al, 1998; Yang et al, 1998; Kaelin, 1999; Prives and Hall, 1999). The *p73L/p63/p51/p40/KET* gene mainly encodes 2 splice variants, p73L/ΔNp63 and p51/TAp63 by different exon usage at its amino terminal regions (see Figure 1). Of the 2, p51 has a transactivation domain at its amino terminus with low identities to those of p73 (30%) and p53 (22%), while p73L completely lacks such a transactivation domain (Augustin et al, 1998; Senoo et al, 1998; Trink et al, 1998; Yang et al, 1998). The putative DNA-binding domain (DBD) of p73L and p51 have high identities to those of p73 (88%) and to p53 (61%). The putative oligomerization domain of p73L and p51 also have high identities to those of p73 (65%) and p53 (37%). Functionally, p51 has been reported to induce growth suppression, apoptosis and up-regulate $p21^{waf1/cip1}$ through

Received 7 September 2000
Revised 15 January 2001
Accepted 25 January 2001

*Correspondence to:* S Habu

GenBank accession number for ΔNp73L: |AB042841.

binding to the p53 regulatory elements (Osada et al, 1998; Yang et al, 1998). In contrast, p73L has been shown to act as a dominant-negative agent to suppress the transactivation activity of both p53 and p51 (Yang et al, 1998). Although p51 is suspected to be a tumour suppressor candidate, it does not seem to be frequently mutated as is the case with *p53* in human cancers (Nishi et al, 1999; Sunahara et al, 1999; Park et al, 2000). However, no evidence concerning the linkage between the expression of these isoforms and human cancers has been documented so far. In this study, we examined various human tumour cell lines as well as primary human cancer specimens to investigate the potential involvement of the *p73L/p63/p51/p40/KET* gene in the pathogenesis of human cancers.

### MATERIALS AND METHODS

### Tissue specimens and cell lines

Tumour samples were obtained with informed consent from 13 patients diagnosed histologically as having skin cancers at Tokai University Hospital and Kitasato University Hospital. All tissues were quickly frozen in liquid nitrogen and stored at – 80˚C until use. 6 normal skin tissues were similarly obtained under surgical treatment. These tissues were processed for preparation of RNA with ISOGEN reagent (Nippon Gene, Tokyo, Japan) according to the manufacturer's instructions. The cell lines used in this study were collected from several institutions as follows. A431 (epidermoid carcinoma), NCI H460 (lung carcinoma), Daudi, K562 (leukemia), DLD1, HCT116, HT29, LoVo, SW480, SW620, WiDR (colon carcinoma), HeLa (cervical carcinoma), LNCap, PC-3 (prostate carcinoma), MCF7, SKBR3 (breast carcinoma) and Saos-2 (osteogenic sarcoma) were obtained from American Type Culture Collection (Rockville, MD). A549, Lu99, SBC3 and SBC5 (lung carcinoma) were obtained from Japanese Cancer Research Resources Bank (Osaka, Japan). Jurkat (leukaemia), ME180 (cervical carcinoma), TE8, TE9, TE10 and TE11 (oesophageal carcinoma) were obtained from the Cell Resource Center for Biomedical Research, Institute of Development, Aging and Cancer, Tohoku University (Sendai, Japan). MKN-1, MKN-74 (stomach carcinoma) and Cos-7 (African green monkey kidney cell line) were obtained from RIKEN Cell Bank (Tsukuba Science City, Japan). Cells were grown in DMEM or RPMI supplemented with 10% fetal bovine serum. The p53 status of each cell line was as follows; p53 wild-type (WT): A549, NCl H460, LoVo, LNCap, HCT116, MCF7, Daudi, SBC3, Lu99, HeLa and ME180; p53 mutant (mt): Saos-2, PC-3, SBC5, WiDR, SKBR3, K562, Jurkat, MKN-1, MKN-74, TE8, TE9, TE10, TE11, A431, SW480, SW620, HT29 and DLD1. Note that the 2 cervical cell lines ME180 and HeLa were grouped with p53WT although they harbour human papilloma viruses (HPV) which inactivate the wild-type p53. Assignment of these 2 cell lines to either group did not make a significant difference in the following statistical analysis.

### RT-PCR/Southern blot analysis

First-strand cDNAs were synthesized from 2 µg of total RNA using cDNA Cycle Kit with random primers (Invitrogen, Carlsbad, CA). The synthesized cDNA was used immediately for PCR amplification or stored at – 20˚C for further analyses. Nested PCR was performed to detect the *p73L/p63/p51/p40/KET* gene transcripts with Mastercycler Gradient (Eppendorf, Hamburg, Germany) with the following primer sets; 5TA or 5dN / 3N1 (1st PCR), 5TA or 5dN / 3N2 (2nd PCR) for detection of p51 and p73L transcripts, respectively, and 5D1 / 3D (1st PCR), 5D2 / 3D (2nd PCR) for detection of the common DNA binding domain (see Figure 1 for the location of the primers). The first amplification was carried out in 25 µl reaction mixture containing 1 µl of RT product through 30 cycles of 94˚C for 40s, 60˚C for 45s, 72˚C for 40s. The second amplification was carried out in 25 µl reaction mixture containing 1 µl of the first PCR product through 35 cycles of the same condition described above. As a control, β-actin cDNA was amplified with 1 µl of RT product through 32 cycles of 94˚C for 40s, 60˚C for 45s, 72˚C for 40s. The oligonucleotide primers used in this study and the expected length of the products are listed in Table 2. The PCR products were run on a 1.2% agarose gel, transferred onto nylon membranes (Pall, Port Washington, NY) and the Southern blots were hybridized with $\gamma$-$^{32}$P labelled oligonucleotide probes: 73L-R2 (indicated as N in Figure 1), 3N2 (indicated as D in Figure 1) and with $\alpha$-$^{32}$P labelled human β-actin cDNA probe (Clontech, Palo Alto, CA) for control β-actin. Bands were exposed to Kodak X-OMAT AR films for 3–4 h.

### Sequence analysis

Nested PCR was performed as described above and the bands were excised from the gel and purified using a QIAquick Gel Extraction Kit (Qiagen, Inc, Santa Clarita, CA). The purified DNA fragments were directly sequenced with both strands by an ABI Prism 373 DNA sequencer (Perkin-Elmer, Foster City, CA) using a Cycle sequencing kit (Perkin-Elmer) according to the manufacturer's protocol. To confirm the presence of mutation, PCR products were blunt-ended and subcloned into the EcoRV site in pBluescript II SK(+) vector (Stratagene, La Jolla, CA) and multiple individual clones were subsequently sequenced on both strands. For genomic sequencing, the intronic primer pairs were used as described previously (Hagiwara et al, 1999).

### Plasmids

The HA-tagged p53 expression vector pcDNA-HAp53 was constructed by inserting the BamHl/Xbal PCR fragment containing the open reading frame of p53 cDNA using pBRBOSwt (Kanda et al, 1994) as a template into pcDNA3 (Invitrogen, Carlsbad, CA). The HA-tagged p73L expression vector was also constructed by PCR-based method using the full length murine p73L cDNA, which we reported previously (Senoo et al, 1998), to generate pcDNA-HAp73L. The HA-tagged p51 expression vector pcDNA-HAp51 was also constructed by RT-PCR followed by HA-tagging from murine testis cDNA. ΔN fragment was PCR-amplified using pcDNA-HAp73L as a template with the following primer pairs: 5′-CGGGATCCATGTTGTACCTGGAAAACAATGCCCAGA-CTCAATTTAGTGAGTATTCCACCGAACTGAAGAAGC-3′ and 5′-AAATTGGACGTCTGTTCATT-3′. The HA-tagged ΔNp73L expression vector pcDNA-HAΔNp73L was constructed by replacement of the BamHl/EcoRl fragment from pcDNA-HAp73L with the HA-tagged ΔN fragment generated by second amplification of ΔN fragment with the following primer pairs: 5′-CGGGATCCATGTACCCATACGATGTTCCAGATTAC-GCTTGTACCTGGAAAACAATG-3′ and 5′-AAATTGGACGT-CTGTTCATT-3′. All the sequences amplified by PCR reaction in

 *© 2001 Cancer Research Campaign*

this study were confirmed as having no mutation by direct sequencing.

### Transient transfections and luciferase assays

For luciferase assay, Cos-7 cells and DLD1 cells grown to ~80% confluence were co-transfected by SuperFect reagent (Qiagen) with the following amounts of expression and reporter plasmids in 24-well culture plates. In Cos-7 cells, a SV40-promoter driven luciferase reporter plasmid pGL2 (0.3 μg, Promega, Madison, Wl) and a p53-responsive luciferase reporter plasmid PG13 (0.3 μg) plus pcDNA-HAp51 (0.6 μg) were co-transfected with certain amounts of pcDNA-HAp73L (0-5 μg) or pcDNA-HAΔNp73L (0-5 μg). In DLD-1 cells, p53-responsive luciferase reporter plasmid PG13 (0.3 μg) plus pcDNA-HAp53 (0.6 μg) or pcDNA-HAp51 (0.6 μg) were co-transfected with increasing amounts of pcDNA-HAp73L (0.6, 1.8 μg) or pcDNA-HAΔNp73L (0.6, 1.8 μg). The activities of *Firefly* luciferase were measured 24–36 hours post transfection using the Dual-Luciferase reporter assay system (Promega). The transfection efficiency was normalized by the *Renilla* luciferase activity by the pRLTK (0.3 μg, Promega) and the relative luciferase activity was calculated in each measurement by the formula: (*Firefly* luciferase activity)/ (*Renilla* luciferase activity).

### RESULTS AND DISCUSSION

### The expression profile of p51/p73L transcripts in human tumour cell lines

The *p73L/p63/p51/p40/KET* gene produces two major isoforms, p51 and p73L, with different amino terminal regions as shown in Figure 1 (see also Table 1). To determine the distinct expression of these two isoforms in tumour cells, we prepared specific oligonucleotide primer sets to amplify each of the amino terminal regions (see Figure 1 for primer locations). Using nested RT-PCR/Southern blot analysis, we examined 29 human tumour cell lines in which the p53 status is well characterized as wild-type or mutant. As summarized in Table 3, the profile of p51/p73L expression was not closely correlated with the p53 status; p51 expression was increased a little in p53 wild-type cell lines in comparison to p53 mutants although statistical analysis showed no significant difference. In addition, tumour cell lines with mutant p53 lacked p51 expression at a high frequency (13/18 cases, 72%), suggesting that p51 and p53 may suppress the same tumour in an independent manner (Figure 2, lanes 7–11, 15, 16, 19–21, 24–26). In regard to p73L, similar results were found between p53 wild-type and p53 mutant cell lines (Table 3). In these 29 tumour cell lines, a high proportion of tumours (27/29, 93%) showed the DNA-binding domain (DBD) which is shared in all isoforms of p73L/p51, but the amino-terminal regions of p73L and p51 were detectable in only 55% (16/29) and 41% (12/29), respectively. Among these cell lines, the dual expression of p73L and p51 was found in only 8 cases out of 29 (28%). It is of note that most colon carcinoma cell lines we examined (SW620, SW480, HT29 and WiDR) and one breast carcinoma cell line, SKBR3, did not show any amino terminal regions corresponding to either p51 or p73L even though they showed robust DBD amplification (Figure 2, lanes 8, 9, 10, 15, 16). These results indicate that the transcription of the *p73L/p63/p51/p40/KET* gene is regulated by multiple promoters or splicing events which presumably generate more isoforms other than those reported previously (Augustin et al, 1998; Osada et al, 1998; Senoo et al, 1998; Trink et al, 1998; Yang et al, 1998). In addition, expression of the *p73L/p63/p51/p40/KET* gene was undetectable in Lu99 (lung carcinoma) and DLD1 (colon carcinoma) (Figure 2, lanes 3 and 11). Southern blot analysis of genomic DNA from these 2 cell lines revealed no gross alterations of this gene when the full-length *p73L* cDNA was used as a probe (data not shown), indicating that the *p73L/p63/p51/p40/KET* gene is transcriptionaly silent in these 2 cell lines.

### A novel truncated isoform of p73L is preferentially expressed in squamous cell lines and primary human squamous carcinomas

In addition to the expected size for p73L and p51, the truncated ΔN-type amino-terminus was detected in several tumour cell lines (Figure 2, lanes 12, 13, 22, 26–29) which were all derived from squamous cells except for LNCap, a prostate-derived tumour cell line. However, such a truncated amino-terminus was not detected in 22 other tumour cell lines including 7 colon-, 2 breast-, 5 lung-, and 2 stomach-carcinomas and 3 leukaemias (Figure 2) even in our high sensitive detection system. By estimating from the size, this truncated band is considered to occur presumably by splicing out

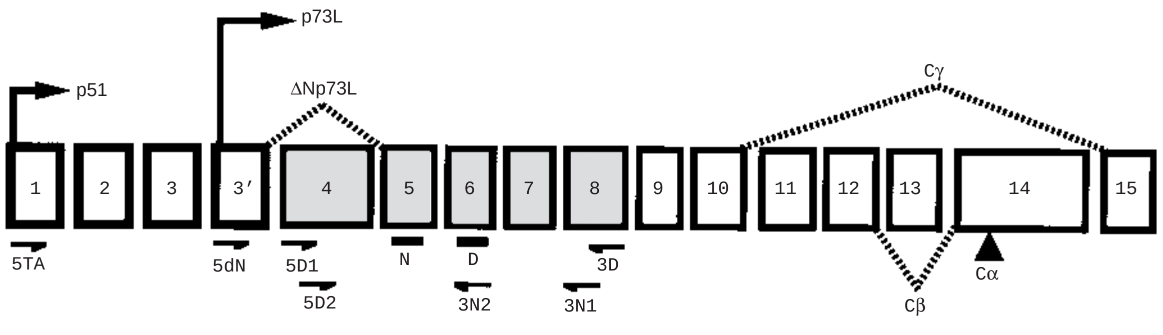

**Figure 1** Schematic representations of the *p73L / p63 / p51 / p40 / KET* gene and the positions of the PCR primer pairs used for RT-PCR / Southern blot analysis. The structure of the *p73L / p63 / p51 / p40 / KET* gene and the major post-transcriptional splicing events giving rise to multiple isoforms are based on a recent study by Yang et al (1998). Exons 4 through 10 are common for all the transcripts (DNA binding domain, grey boxes). Exons 1 through 3 encode the transactivation domain of p51, while p73L is encoded by exon 3′ at its amino terminus. The α-type carboxy terminus is encoded by the exons through 14, and the position of the termination codon is indicated by a solid triangle. In contrast, β- and γ-type carboxy termini lack exons 13 and 11 through 14, respectively. A novel isoform, termed ΔNp73L, which lacks exon 4 from the primary p73L transcript, is also indicated by a dashed line (see text for details). 5′ primers 5TA and 5dN are the specific primers in the amino terminal regions of p51 and p73L, respectively. Note that ΔNp73L can be amplified with the same primer sets as used for p73L amplification. The primers in the common region among the isoforms are also indicated (3N1, 3N2, 5D1, 5D2 and 3D). The Southern blots were hybridized with the specific oligonucleotide probes shown in solid bars; probes N and D for the amino termini and the DNA binding domain, respectively

© 2001 Cancer Research Campaign

**Table 1** The *p73L / p63 / p51 / p40 / KET* gene isotypes

| Isotypes | Synonyms | Amino acid length | Exon usage |
|---|---|---|---|
| p51 | TAp63α / p51B / KET | 641 | 1–14 |
| | TAp63β | 516 | 1–12, 14 |
| | TAp63γ / p51A | 448 | 1–10, 15 |
| p73L | ΔNp63α | 586 | 3′, 4–14 |
| | ΔNp63β | 461 | 3′, 4–12, 14 |
| | ΔNp63γ | 393 | 3′, 4–10, 15 |
| | p40 | 356 | 3′, 4–10 |
| ΔNp73L | ΔNp73Lα | 501 | 3′, 5–14 |
| | ΔNp73Lβ | 376 | 3′, 5–12, 14 |
| | ΔNp73Lγ | 308 | 3′, 5–10, 15 |

Major isoforms p51 and p73L as well as newly found ΔNp73L in this study, their synonyms, amino acid length and the exon usage are summarized. p51 and p73L start its translation from exon 1 and 3′, respectively, and ΔNp73L is spliced out exon 4 from p73L

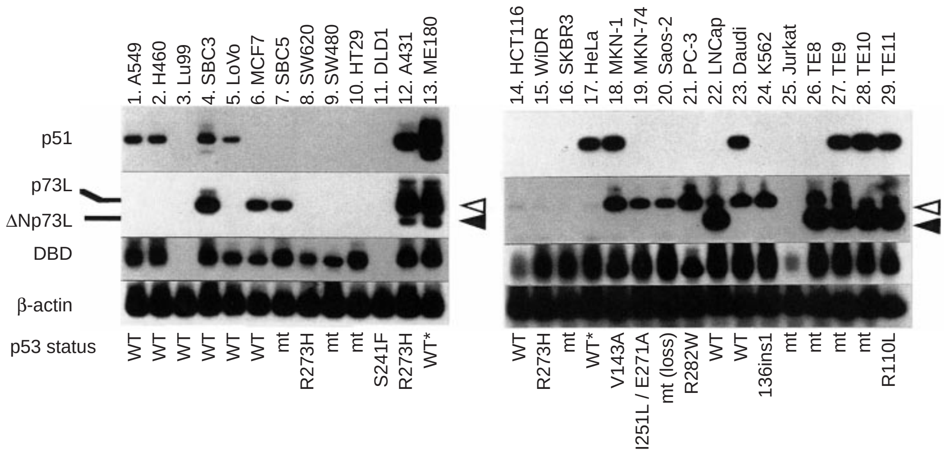

**Figure 2** Detection of the *p51 / p73L* transcripts in various human tumour cell lines by RT-PCR followed by Southern hybridization. Total RNAs were extracted from 29 human tumour cell lines, and 2 amino termini for p51 and p73L and the common DNA-binding domain (DBD) among all the isoforms were amplified by nested RT-PCR, electrophoresed in 1.2% agarose gel, and transferred to nylon membranes followed by hybridization with the specific oligonucleotide probes as described in ‘Materials and Methods’. Details concerning the primer sequences and the expected length of the products are listed in Table 2. The solid and open triangles on the right of each panel indicate the ΔNp73L and p73L, respectively. RNA quality was confirmed by amplification of a β-actin amplicon (lowest panel). The p53 status in each cell line is indicated at the bottom of the panels. *Note that the two cervical cell lines ME180 and HeLa were grouped with p53WT in this study, although they harbor oncoproteins encoded by human papilloma viruses, which inactivate wild-type p53

**Table 2** Nucleotide sequences of the primers used for nested RT-PCR and the expected length of the products

| Primer[a] | Nucleotide sequence | Product size |
|---|---|---|
| 5D1 (1st sense) | 5′- CTCCTGAACAGCATGGACCAGC - 3′ | 780 bp |
| 5D2 (2nd sense) | 5′- ACAGTTCCGACGTGTCCTTC - 3′ | 590 bp |
| 3D (antisense) | 5′- CTTCGTACCATCACCGTTCTT - 3′ | |
| 5TA (sense) | 5′- ATGAATTCCTCAGTCCAGAGG - 3′ | 990/720 bp[b] |
| 5dN (sense) | 5′- TGCCCAGACTCAATTTAGTG - 3′ | 800/530 bp[b] |
| 3N1 (1st antisense) | 5′- CTGTCCGAAACTTGCTGCTT - 3′ | |
| 3N2 (2nd antisense) | 5′- GGATCTTCTACATACTGGGC - 3′ | |

[a] Products generated with primers 5TA and 5dN correspond to p51 and p73L, respectively, and ΔNp73L is detectable by the same primer pairs used for p73L (see Figure 1 for details). [b]The expected length of the product is indicated as 1st PCR product/2nd PCR product.

exon 4 of the *p73L* primary transcript without a frameshift, which was confirmed by direct sequencing of the isolated cDNA fragments (data not shown). Then, we named this novel truncated isoform ‘ΔNp73L’.

To determine whether this ΔNp73L is expressed in normal squamous cells or specifically in tumours, we examined its expression profile in human squamous cell carcinomas from the skin and in the corresponding normal skin tissues. As shown in Figure 3, ΔNp73L

 *© 2001 Cancer Research Campaign*

**Table 3** Expression incidence of p51, p73L, ΔNp73L as well as the common DNA binding domain (DBD) of p51, p73L and ΔNp73L in the p53 wild-type (WT) and p53 mutant (mt) cell lines

| | Total (*n* = 29) | p53WT (*n* = 11) | p53mt (*n* = 18) | $\chi^2$- test |
|---|---|---|---|---|
| DBD | 27 (94%) | 10 (91%) | 17 (94%) | |
| p51 | 12 (41%) | 7 (64%) | 5 (28%) | $P < 0.10$ |
| p73L | 16 (55%) | 5 (46%) | 11 (61%) | $P < 0.50$ |
| ΔNp73L | 7 (24%) | 2 (18%) | 5 (28%) | $P < 0.75$ |

was detected only in squamous cell carcinomas (5/13, 39%, middle panel, lanes 8, 9, 11, 17, 19) but never detectable in normal skin tissues in our highly sensitive detection system (middle panel, lanes 1–6). In addition, we did not detect this novel isoform in the other primary tissue specimens such as 16 stomach-, 19 breast-, 10 ovarian-cancers as well as the corresponding normal samples (data not shown). These results strongly suggest that the expression of ΔNp73L is highly associated with the carcinogenesis of skin tissue. On the other hand, while p51 was detected in all normal skin tissues, its expression in the carcinomas was found at a relatively low frequency (4/13, 31%). Among these p51-expressing squamous cell carcinomas, however, the expression of ΔNp73L was highly associated (3/4, 75%), while the majority of p51-lacking tumours were found not to express ΔNp73L (7/9, 78%). Similarly, most of the squamous cell lines expressing ΔNp73L also expressed p51 but lacked functional p53 (Figure 2, lanes 12, 27–29). From these data, it can be speculated that if ΔNp73L has a dominant-negative function as p73L does, p51 is the more preferable target of ΔNp73L than p53. At the same time, it is also possible to speculate that the dual expression of p73L and ΔNp73L may strengthen the tumorigenesis in p51-expressing tissues.

### ΔNp73L possesses dominant-negative activity against p53 and p51

Since ΔNp73L still retains the DNA-binding domain (see Figure 1), we then examined whether ΔNp73L shares dominant-negative activities with the authentic p73L against both p53 and p51. To address this issue, we transfected DLD1 cells with a constant amount (0.6 μg) of wild-type p53 (Figure 4A, left panel) and p51 (right panel) and varying amounts (0.6 or 1.8 μg) of either p73L (columns 3, 4, 9 and 10) or ΔNp73L (columns 5, 6, 11 and 12) as indicated and assayed for transactivation of the p53-responsive reporter gene. The dominant-negative activity of ΔNp73L against p53 was slightly weaker than that of p73L (Figure 4A, compare columns 5 and 6 to 3 and 4) but was almost equivalent in a dose-dependent inhibition of p51 transactivation to the authentic p73L (Figure 4A, compare columns 11 and 12 to 9 and 10), with the higher suppressor concentration (1.8 μg) giving an almost background-like (empty vector) level of reporter signal (Figure 4A, compare column 12 to 7). The luciferase activity driven by SV40-promoter was unaffected at around this concentration of expression vector (Figure 4B, 0-2 μg of expression plasmid). These results suggest that ΔNp73L specifically suppresses the transcription driven by the p53 / p51-responsive promoter. These data may also imply that the dominant-negative activity of ΔNp73L targets p51 more preferably than p53 (Figure 4A, compare columns 2 and 5 to 8 and 11) compared to the equivalent suppression by p73L on p53- and p51- transactivation (Figure 4A, compare columns 2 and 3 to 8 and 9).

Though mutations in the *p53* gene have been found mostly in its DNA-binding domain (Greenblatt et al, 1994; Hainaut et al, 1998), there was no mutation or frameshift in the DNA-binding domain of the *p73L/p63/p51/p40/KET* gene in the squamous cell carcinomas we examined (data not shown). In contrast, we found the loss of p51 expression and the expression of a novel isoform, ΔNp73L, in the squamous carcinomas. Thus, transcriptional dysregulation of this gene may play a more important role(s) than inactivation of p51 itself by somatic mutations in the tumorigenesis of squamous cells. From this point of view, it is also assumed that the inactivation machinery of the putative tumour suppressor p51 differs from that of p53.

In contrast to p51 and ΔNp73L, the expression of p73L was constitutively detected in all the normal and cancerous specimens we examined (Figure 3, middle panel, lanes 1–19). On the other hand, Parsa et al have recently shown that expression of p73L is gradually decreased during the terminal differentiation of squamous cells (Parsa et al, 1999). As for this discrepancy, we can speculate that detection of the p73L transcript in the tumour specimens we examined is saturated in our system due to its high sensitivity. Since the cancerous specimens we examined were biopsied at a relatively early stage of tumorigenesis, it would be interesting to examine the expression profile of ΔNp73L / p73L / p51 in highly differentiated squamous carcinomas. Whatever the reason, in order to clarify the biological significance of ΔNp73L in tumours expressing a certain amount of p73L, a more quantitative determination of p73L / ΔNp73L at the protein level will be required.

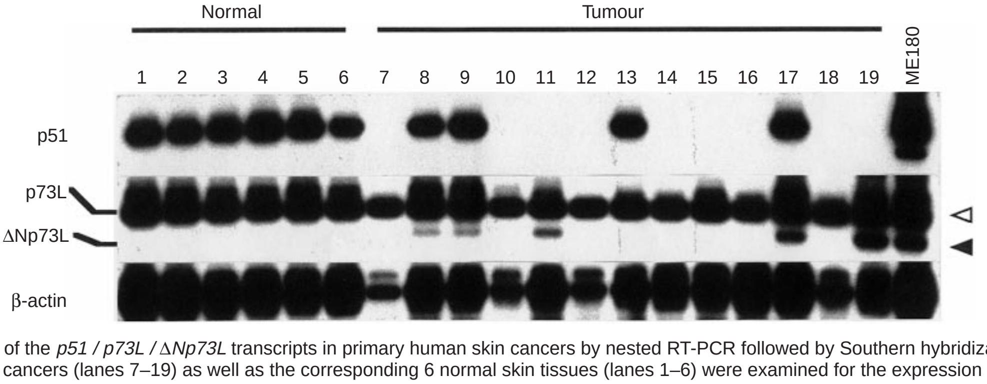

**Figure 3** Detection of the *p51 / p73L / ΔNp73L* transcripts in primary human skin cancers by nested RT-PCR followed by Southern hybridization. Total RNAs from 13 primary skin cancers (lanes 7–19) as well as the corresponding 6 normal skin tissues (lanes 1–6) were examined for the expression profile of the p51 / p73L / ΔNp73L transcripts. The solid triangle on the right (middle panel) indicates the truncated ΔN-type amino termini named ΔNp73L, while the open triangle indicates the authentic p73L. Note that among cancerous specimens, the expression of p51 was absent in 69% (9 cases out of 13), while ΔNp73L was expressed in 39% (5 cases out of 13). A cervical carcinoma cell line, ME180, was used as a positive control for each amplification. Products of RT-PCR amplification of β-actin served as RNA quality control (lower panel)

© 2001 Cancer Research Campaign 

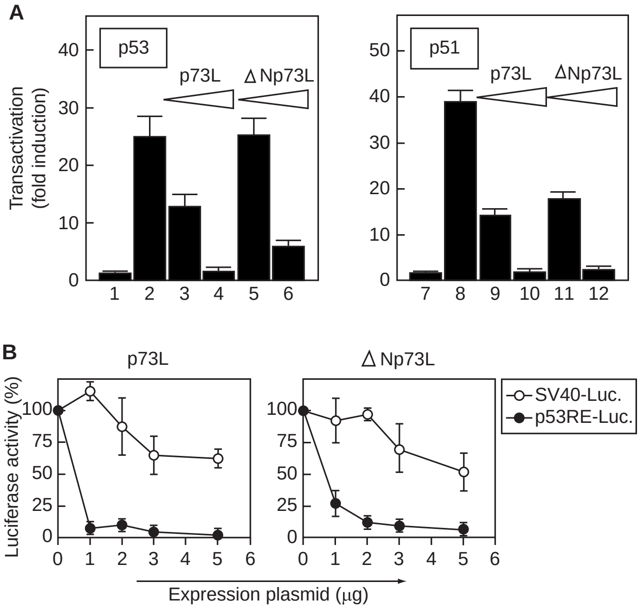

**Figure 4** The novel isoform ΔNp73L acts in a dominant-negative manner toward both p53 and p51 in mammalian cells. (**A**) Exponentially growing DLD1 cells were transiently co-transfected by lipofection with 0.6 μg of the p53 expression vector (left panel, columns 2–6) or the p51 expression vector (right panel, columns 8–12) together with 0.3 μg of PG13 in the presence of increasing amounts (0.6 μg or 1.8 μg) of either p73L expression vector (lanes 3, 4, 9 and 10) or ΔNp73L expression vector (lanes 5, 6, 11 and 12) as indicated. Lanes 1 and 7 were the results from mock transfectants (empty vector). The data represent the average of 3 independent experiments. (**B**) Exponentially growing Cos-7 cells were transiently co-transfected by lipofection with 0.3 μg of the SV40 promoter-luciferase gene (pGL2, open circles) or the p53-responsive luciferase gene (PG13, closed circles) plus 0.6 μg of the p51 expression vector in the presence of increasing amounts of either p73L expression vector (0-5 μg, left panel) or ΔNp73L expression vector (0–5 μg, right panel) as indicated. Results are shown as relative luciferase activity to control cells transfected with an empty vector. The data represent the average of 3 independent experiments

In summary, we have shown that transcription of the *p73L/p63/p51/p40/KET* gene is dysregulated in human squamous cell carcinomas of the skin. A novel dominant-negative isoform, termed ΔNp73L, was detected in squamous cell carcinoma cell lines as well as in primary human skin cancer specimens but not in the normal skin tissues. Conversely, the expression of the potential tumour suppressor p51 was frequently lost in the cancerous specimens we examined. Furthermore, our present study using human tumour cell lines indicated that the *p73L/p63/p51/p40/KET* gene may be expressed independently of the p53 status. From these results, we hypothesize that the *p73L/p63/p51/p40/KET* gene might contribute to tumorigenesis in human cancers through its altered expression. To define the oncogenic properties of p73L/ΔNp73L and the tumour suppressive properties of p51, further investigations will be required. Revealing the down-stream target genes of p51 / p73L and the epigenetic modification mechanisms of the *p73L/p63/p51/p40/KET* gene will help to gain a better understanding of the dual roles of this gene in tumour biology.

### ACKNOWLEDGEMENTS

We thank Drs T Tajima, H Maku-uchi (Tokai University Hospital) and M Masuzawa (Kitasato University Hospital) for providing tissue specimens, Drs K Segawa (Keio University) and B Vogelstein (Johns Hopkins University) for providing the plasmids pBRBOSwt and PG13, respectively, and Mr A Gerz for reading the manuscript.

### REFERENCES

Augustin M, Bamberger C, Paul D and Schmale H (1998) Cloning and chromosomal mapping of the human p53-related *KET* gene to chromosome 3q27 and its murine homolog Ket to mouse chromosome 16. *Mamm Genome* **9**: 899–902

Di Como CJ, Gaiddon C and Prives C (1999) p73 function is inhibited by tumor-derived p53 mutants in mammalian cells. *Mol Cell Biol* **19**: 1438–1449

Dobbelstein M, Wienzek S, Konig C and Roth J (1999) Inactivation of the p53-homologue p73 by the mdm2-oncoprotein. *Oncogene* **18**: 2101–2106

Greenblatt MS, Bennett WP, Hollstein M and Harris CC (1994) Mutations in the *p53* tumor suppressor gene: clues to cancer etiology and molecular pathogenesis. *Cancer Res* **54**: 4855–4878

Hagiwara K, McMenamin MG, Miura K and Harris CC (1999) Mutational analysis of the *p63 / p73L / p51 / p40 / CUSP / KET* gene in human cancer cell lines using intronic primers. *Cancer Res* **59**: 4165–4169

Hainaut P, Hernandez T, Robinson A, Rodriguez-Tome P, Flores T, Hollstein M, Harris CC and Montesano R (1998) IARC database of p53 gene mutations in human tumors and cell lines: updated compilation, revised formats and new visualisation tools. *Nucleic Acids Res* **26**: 205–213

Hollstein M, Sidransky D, Vogelstein B and Harris CC (1991) p53 mutations in human cancers. *Science* (Washington DC) **253**: 49–53

Jost CA, Marin MC and Kaelin WG Jr (1997) p73 is a human p53-related protein that can induce apoptosis. *Nature* **389**: 191–194

Kaelin WG Jr (1999) The emerging p53 gene family. *J Natl Cancer Inst* **91**: 594–598

Kaghad M, Bonnet H, Yang A, Creancier L, Biscan JC, Valent A, Minty A, Chalon P, Lelias JM, Dumont X, Ferrara P, McKeon F and Caput D (1997) Monoallelically expressed gene related to p53 at 1p36, a region frequently deleted in neuroblastoma and other human cancers. *Cell* **90**: 809–819

Kanda T, Segawa K, Ohuchi N, Mori S and Ito Y (1994) Stimulation of polyomavirus DNA replication by wild-type p53 through the DNA-binding site. *Mol Cell Biol* **14**: 2651–2663

Laurenzi VD, Costanzo A, Barcaroli D, Terrinoni A, Falco M, Annicchiarico-Petruzzelli M, Levrero M and Melino G (1998) Two new p73 splice variants, γ and δ, with different transcriptional activity. *J Exp Med* **188**: 1763–1768

Levine AJ, Momand J and Finlay CA (1991) The *p53* tumour suppressor gene. *Nature* (Lond.) **351**: 453–456

Marin MC, Jost CA, Irwin MS, DeCaprio JA, Caput D and Kaelin WG Jr (1998) Viral oncoproteins discriminate between p53 and the p53 homolog p73. *Mol Cell Biol* **18**: 6316–6324

Nishi H, Isaka K, Sagawa Y, Usuda S, Fujito A, Ito H, Senoo M, Kato H and Takayama M (1999) Mutation and transcription analyses of the p63 gene in cervical carcinoma. *Int J Oncol* **15**: 1149–1153

Osada M, Ohba M, Kawahara C, Ishioka C, Kanamaru R, Katoh I, Ikawa Y, Nimura Y, Nakagawara A, Obinata M and Ikawa S (1998) Cloning and functional analysis of human *p51*, which structurally and functionally resembles *p53*. *Nature Med* **4**: 839–843

Park B-J, Lee S-J, Kim JI, Lee SJ, Lee C-H, Chang S-G, Park J-H and Chi S-G (2000) Frequent alteration of p63 expression in human primary bladder carcinomas. *Cancer Res* **60**: 3370–3374

Parsa R, Yang A, McKeon F and Green H (1999) Association of p63 with proliferative potential in normal and neoplastic human keratinocytes. *J Invest Dermatol* **113**: 1099–1105

Prabhu NS, Somasundaram K, Satyamoorthy K, Herlyn M and El- Deiry WS (1998) p73, unlike p53, suppresses growth and induces apoptosis of human papillomavirus E6-expressing cancer cells. *Int J Oncol* **13**: 5–9

Prives C and Hall PA (1999) The p53 pathway. *J Pathol* **187**: 112–126

Schmale H and Bamberger C (1997) A novel protein with strong homology to the tumor suppressor p53. *Oncogene* **15**: 1363–1367

Senoo M, Seki N, Ohira M, Sugano S, Watanabe M, Inuzuka S, Okamoto T, Tachibana M, Tanaka T, Shinkai Y and Kato H (1998) A second p53-related protein, p73L, with high homology to p73. *Biochem Biophys Res Commun* **248**: 603–607

Steegenga WT, Shvarts A, Riteco N, Bos JL and Jochemsen AG (1999) Distinct regulation of p53 and p73 activity by adenovirus E1A, E1B, and E4orf6 proteins. *Mol Cell Biol* **19**: 3885–3894

Sunahara M, Shishikura T, Takahashi M, Todo S, Yamamoto N, Kimura H, Kato S, Ishioka C, Ikawa S, Ikawa Y and Nakagawara A (1999) Mutational analysis of p51A / TAp63γ, a p53 homolog, in non-small cell lung cancer and breast cancer. *Oncogene* **18**: 3761–3765

Trink B, Okami K, Wu L, Sriuranpong V, Jen J and Sidransky D (1998) A new human p53 homologue. *Nature Med* **4**: 747

Ueda Y, Hijikata M, Takagi S, Chiba T and Shimotohno K (1999) New p73 variants altered C-terminal structures have varied transcriptional activities. *Oncogene* **18**: 4993–4998

Weinberg R (1993) Tumor suppressor genes. *Neuron* **11**: 191–196

*© 2001 Cancer Research Campaign*

Yang A, Kaghad M, Wang Y, Gillett E, Fleming MD, Dotsch V, Andrews NC, Caput D and McKeon F (1998) *p63*, a *p53* homolog at 3q27–29, encodes multiple products with transactivating, death-inducing, and dominant-negative activities. *Mol Cell* **2**: 305–316

Zeng X, Chen L, Jost CA, Maya R, Keller D, Wang X, Kaelin WG Jr, Oren M, Chen J and Lu H (1999) MDM2 suppresses p73 function without promoting p73 degradation. *Mol Cell Biol* **19**: 3257–3266

*© 2001 Cancer Research Campaign*
